# Let's get high: Cladogenesis in freshwater crabs (Decapoda: Potamonautidae: *Potamonautes*) supports the mountain gradient speciation hypothesis in the Cape Fold and Drakensberg Mountains, South Africa

**DOI:** 10.1002/ece3.10960

**Published:** 2024-03-05

**Authors:** Savel R. Daniels, Nasreen Peer, Angus Macgregor Myburgh, Aaron Barnes, Sebastian Klaus

**Affiliations:** ^1^ Department of Botany and Zoology University of Stellenbosch Stellenbosch South Africa; ^2^ Department of Ecology and Evolution J. W. Goethe‐University, Biologicum Frankfurt am Main Germany; ^3^ ERM GmbH Neu‐Isenburg Germany

**Keywords:** aquatic, biogeography, diversity, refugia, speciation

## Abstract

During the present study, the evolutionary relationship within a clade of mountain clade of freshwater crabs (*Potamonautes*) was examined using mtDNA sequence data for species from the Cape Fold Mountain (CFM) and Great Escarpment (Drakensberg Mountain range). We undertook phylogenetic analyses, divergence time estimation, and an ancestral area reconstruction to explore the period of cladogenesis and understand the biogeographic history in this high‐altitude clade. Furthermore, we applied four species delimitation methods using ASAP, bPTP, bGMYC, and STACEY on the latter clade. Bayesian phylogenetic analyses retrieved a monophyletic freshwater crab clade comprised of two major sister clades, one comprised of the Cape Fold (clade A) and two comprised of Drakensberg Mountains (clade B) species. Divergence time estimation indicated that the two clades underwent Mio/Pliocene cladogenesis. Within the CFM clade (A), *P. amathole* (Amathola Mountains) was sister to *P. parvispina* (Cederberg and Kouebokkeveld Mountains) and the latter species were sister to *P. parvicorpus* (Cape Peninsula, Jonkershoek, and Helderberg Mountains) sister to *P. tuerkayi* (Overberg Mountains) and *P. brincki* (Hottentots Holland Mountains). Within the Drakensberg Mountain clade (B), we observed *in situ* diversification. Specimens from the southcentral Drakensberg Mountains (Dargle Forest, Injasuti, Karkloof, and Impendle) represent a new undescribed lineage *Potamonautes* sp. nov. 1. The second clade from the northern Drakensberg, representing *P. clarus*, was sister to a central Drakensberg Mountain clade that comprised *P. depressus* that was in turn sister to *P. baziya* from the Eastern Cape Province. The application of species delimitation methods generally overestimated the number of species. The biogeographic analyses indicated that the Eastern Cape Province is the most likely ancestral range area. Ecological niche modelling of representative species in clades A (Cape Fold Mountains) and B (Drakensberg Mountains) demonstrated that temperature and rainfall were the major abiotic drivers that differentiated the two clades. Our data favours the mountain gradient speciation hypothesis.

## Graphical abstract

BioGeoBears analyses of the biogeographic analyses in a endemic clade of freshwater crabs in South Africa. The figure includes the tree topology and a picture of the Cape Fold Mountains and the Drakensberg Mountains.

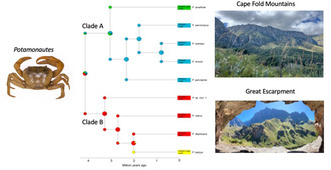

## 1 INTRODUCTION

Mountains are considered reservoirs for biodiversity and are characterised by exceptional levels of faunal and floral endemicity. The complex interactions between abiotic and biotic factors coupled with life history characteristics are responsible for spatial and temporal dynamics of cladogenesis in mountain living taxa (Mosbrugger et al., 2018; Muellner‐Riehl, 2019; Oswald et al., 2022; Pujalor et al., 2022). Abiotic factors impacting evolutionary diversification in mountain inhabiting species include tectonic uplift, edaphic heterogeneity, ancient and recent climatic oscillations (with temperature and rainfall being central), altitudinal gradients, intensity of glacial and interglacial periods, and erosion cycles (chemical and mechanical weathering) (Couvreur et al., 2020). Biotic factors include habitat discontinuity, the ecological niche being occupied, dispersal capability of the species, physiological tolerance, and competitive interaction between species (Fjeldsã et al., 2012; Habel et al., 2015; Voelker et al., 2010; Voje et al., 2009). The interaction between these abiotic and biotic factors sets the arena for natural selection, adaptation, and cladogenesis in mountain dwelling taxa.

Two opposing evolutionary hypotheses exist to explain the high faunal and floral diversity in mountainous regions. The mountain‐geobiodiversity hypothesis (MGH) proposes that the steep ecological gradients present on mountains potentially allows for adaptation and ecological speciation, with climatic cycles resulting in fragmentation driving allopatric cladogenesis with low risks of extinction because of the elevational shifts that species can use to compensate for temperature variability (Mosbrugger et al., 2018; Muellner‐Riehl, 2019). These attributes allow mountainous regions to act as refugia and cradles of speciation, sustaining ancient lineages. Under the MGH, sister species co‐occur on the same mountain block have non‐overlapping elevation ranges, and cladogenesis is linked to mountain orogeny. By contrast, the mountain gradient speciation hypothesis (MGSH) links cladogenesis to climatic ameliorations via vicariance as opposed to ecological/adaptation induced processes (Voelker et al., 2010; Voje et al., 2009). Under the MGSH, speciation should be closely correlated with periods of climatic fluctuations. In addition, sister species with overlapping altitudinal ranges occur in allopatry on different mountain ranges under this hypothesis.

Globally, tropical mountain ranges represent well‐studied exemplars of evolutionary biology (Chazot et al., 2017; Fjeldsã et al., 2012; Habel et al., 2015; Oswald et al., 2022; Pujalor et al., 2022). Classic evolutionary studies exist in the Neotropics, where focal research on Andean cordillera taxa revealed the impact of discontinuous habitat on the phylogenetic structure of species, demonstrating that geographically adjacent species are not necessarily sister species, further revealing a complex non‐linear and intricate web of colonisation and migration (Benham & Witt, 2016; Hurtado & d'Elía, 2021; Winger et al., 2015). Similarly, Afrotropical fauna in the Eastern Arch ‘sky island’ mountains along the Great Rift Valley systems comprise well‐studied models of speciation (Fjeldsã et al., 2012; Habel et al., 2015). These mountain massifs differ in geological age, altitude, size, and geographic isolation and are frequently bisected by habitat fragmentation promoting reproductive isolation. Tectonic activity, initiated during the Plio/Pleistocene, was a major catalyst driving evolutionary diversification. The corollary is that vicariant events are central in the development of genetic isolation, resulting in species‐rich endemic clades. Most evolutionary studies in mountainous regions have focused on tropical vertebrate fauna, in particular avifauna, small mammals, and reptiles (Benham & Witt, 2016; Fjeldsã et al., 2012; Habel et al., 2015; Hurtado & d'Elía, 2021; Winger et al., 2015). In contrast, the evolutionary drivers of diversification among mountain dwelling invertebrates in temperate regions are poorly explored. Examining patterns of speciation in temperate mountain ranges may yield novel insight into specific mechanisms of speciation. For example, organisms on temperate mountains have broader thermal niches when compared to tropical taxa, suggesting that temperature profiles might be less important as a mechanism for divergence. Consequently, the study of evolution in temperate mountain regions is likely to provide insight into patterns and processes responsible for generating local biodiversity, especially in light of predicted climate change.

Two mountain types dominate the South African topography, the Cape Fold Mountains (CFM), and the Great Escarpment (GE). The CFM are a series of parallel ranges that run across south–north and south‐easterly axes parallel to the coast from the Cederberg Mountains to the Cape Peninsula in the Western Cape province and southwards towards Gqeberha (formerly Port Elizabeth) in the Eastern Cape province (Blewett & Phillips, 2015; Cowling et al., 2009). The north–south axes border the cold Atlantic Ocean, while the south‐easterly axes border the warm Indian Ocean. The close geographic proximity of the CFM to the two distinct ocean basins likely provides some moderation from climatic fluctuations. However, dramatic ancient shifts in oceanic currents along the west (the cold Benguela current) and southeast coasts (the warm Agulhas current) impacted climatic regimes in the terrestrial domain by altering temperature and precipitation, thus promoting habitat perturbations (Cowling et al., 2009; Siesser, 1980). The CFM are Palaeozoic in age and are characterised by extensive erosion and deeply incised intermountain valleys and coastal planes, with several massifs being geographically isolated (Deacon et al., 1992). Along the north–south axes, mountains are higher and steeper in relief compared the east. The dramatic CFM are moderate in altitude, with considerable variability, the absence of foothills rising directly from sea level or the valley floors. Following a period of tectonic stability in the CFM, uplift occurred during the Miocene epoch (Deacon et al., 1992). Tectonic uplift was again initiated during the Pliocene and resulted in a similar uplift pattern as observed during the Miocene, promoting rampant river capture. These orographic events significantly modified the topography of the region. The west of the CFM comprises a winter rainfall region, while the south‐eastern portions are generally regarded to fall into a transitional rainfall area. These rainfall regimes have been established since the Pliocene (Deacon et al., 1992; Tyson & Partridge, 2000). The CFM forms the boundary of the Greater Cape Floristic region, and encompasses two biodiversity hotspots, the fynbos biome (a Mediterranean heathland) and the Succulent‐Karoo, both renowned for their spectacular floristic diversity and endemism (Mittermeier et al., 2005).

The Great Escarpment (GE) in southern Africa extends from the borders of Mozambique and Zimbabwe into South Africa, Eswatini (formerly Swaziland), and Lesotho, extending further into Namibia and Angola. The escarpment is approximately 5000 km long, semi‐continuous, and consists of steep slopes that frequently face the ocean, while geomorphologically it exists as an extensive plateau rim (Clark et al., 2011a, 2011b). In South Africa, the GE starts in the Limpopo province, extending south into Mpumalanga, KwaZulu‐Natal, and the Eastern Cape province, west towards the Western Cape province where it runs inland in parallel to the CFM, and finally terminates in the Northern Cape province. The GE is an area of spectacular floral and vertebrate endemism (Clark et al., 2011a). The Drakensberg Mountains, comprising the easterly portion of the GE extending north‐eastwards reach an elevation of >3400 m, with near vertical scarps up to 600 m high. As a consequence of its relief, temperature and precipitation regimes are highly variable. The Drakensberg Mountains are ancient and estimated to be in the region of 180 Mya (Hargreaves et al., 1997). Similar to the CFM, the Drakensberg Mountains experienced two recent uplifts during the early Mio/Pliocene (Cowling et al., 2009). The Drakensberg Mountains are characterised by summer rainfall, a pattern that has been established since the Plio/Pleistocene. In contrast to the CFM, the Drakensberg Mountains experienced marked Holocene temperature increases and more mesic periods, altering floral and faunal composition patterns during the Plio/Holocene. Dated phylogenies for the Drakensberg Mountain inhabiting taxa suggest that pulses of diversification are strongly linked to Plio/Pleistocene uplift and the subsequent erosion of the landscape (Bentley et al., 2014; Sands et al., 2022).

Phylogenetic evidence exists for a paleo‐connectivity between fauna and flora from the CFM and the GE (Clark et al., 2011b). However, this remains poorly understood in faunal groups and a limited number of evolutionary studies have been conducted in South Africa to explore mechanisms of temperate mountain speciation. Evolutionary studies of mountain dwelling invertebrate lineages are few, hampering our understanding of patterns and processes promoting cladogenesis in the region. The southern African freshwater crab fauna, *Potamonautes*, is well studied, and the taxonomy of the genus is considered stable (Cumberlidge & Daniels, 2022). New freshwater crab species are constantly being discovered in the region, suggesting considerable hitherto unsampled diversity due to morphologically cryptic species and poorly sampled areas (Cumberlidge & Daniels, 2022; Daniels et al., 2023; Peer et al., 2023). Phylogenetically, two major freshwater crab clades can be discerned; a monophyletic group of temperate small‐bodied mountain‐dwelling species, and its sister clade of large‐bodied riverine, subtropical and temperate species (Daniel et al., 2021; Daniels et al., 2002, 2006, 2015). Within the mountain clade, two clades can be further discerned, a clade comprised exclusively of five first‐order mountain stream species endemic to the CFM, sister to a clade of two species endemic to the Drakensberg Mountains along the GE (Daniels et al., 2002, 2003, 2006, 2015). The CFM freshwater crab clade species occur in allopatry on distinct CFM blocks in the Eastern and Western Cape provinces (Daniels et al., 2023). In contrast, the Drakensberg Mountain clade is comprised of two described and several cryptic freshwater crab lineages, on the same range (Daniels et al., 2003; Phiri & Daniels, 2016). Furthermore, the Drakensberg Mountain clade appears to be the result of in situ diversification and is sister to *P. baziya* occurring on the GE in the Eastern Cape province, South Africa (Daniels et al., 2003; Phiri & Daniels, 2016). Alpha taxonomic diversity within the Drakensberg Mountain freshwater crab clade remains dubious, poorly quantified, and forms the ideal template with which to test the utility of recently developed species delimitation methods. The recent discovery of two new mountain species (*P. amathole* and *P. baziya*) in the intermediary area in the Eastern Cape province (each belonging to the CFM and the Drakensberg Mountain clades, respectively) can facilitate novel insight into the mechanics of speciation between the two geologically distinct mountainous regions (Daniel et al., 2021; Peer et al., 2023). To date, the freshwater crab fauna of the CFM have remained devoid of fine‐scale sampling, and the evolutionary relationship with the Drakensberg Mountain fauna is poorly explored. Combining existing and new mtDNA sequence data for both the CFM and the Drakensberg Mountains can illuminate the evolutionary drivers responsible for cladogenesis in temperate mountains and provide corroborative evidence for either the MGH or the MGSH.

During the present study, we examine the fine‐scale evolutionary relationship between the CFM and Drakensberg Mountains freshwater crab species to test which of the two competing hypotheses (MGH or MGSH) best explain the observed cladogenesis, and how the CFM and GE species are phylogenetically linked. We undertook a divergence time estimation on the phylogenetic topology and employ ancestral area reconstruction to explore the colonisation history of the CFM and the Drakensberg Mountains. We posed the following four research questions: What is the directionality of colonisation for the South African mountain freshwater crabs? Did unidirectional dispersal from the CFM freshwater crab fauna result in the colonisation of the Drakensberg Mountains or vice versa? Was cladogenesis driven exclusively by climatic ameliorations or vicariance events or a combination of these factors? How many species are present specifically in the Drakensberg Mountain clade as evident from the application of species delimitation methods? In addition, we also undertook niche modelling on the distribution of the two major clades to determine the degree of niche overlap between the two clades, as well as to determine the major climatic variables influencing their respective distributions. We hypothesise that climatic ameliorations in South Africa during the Plio/Pleistocene drove cladogenesis in the group, and that diversification within the CFM and the Drakensberg Mountains reflects the impact of further climatic deterioration during the Plio/Pleistocene, suggesting that that speciation is decoupled from orographic uplift. Furthermore, we hypothesise *a priori* that the MGSH will be supported by our data.

## 2 MATERIALS AND METHODS

### 2.1 Sample collection

**FIGURE 1:**
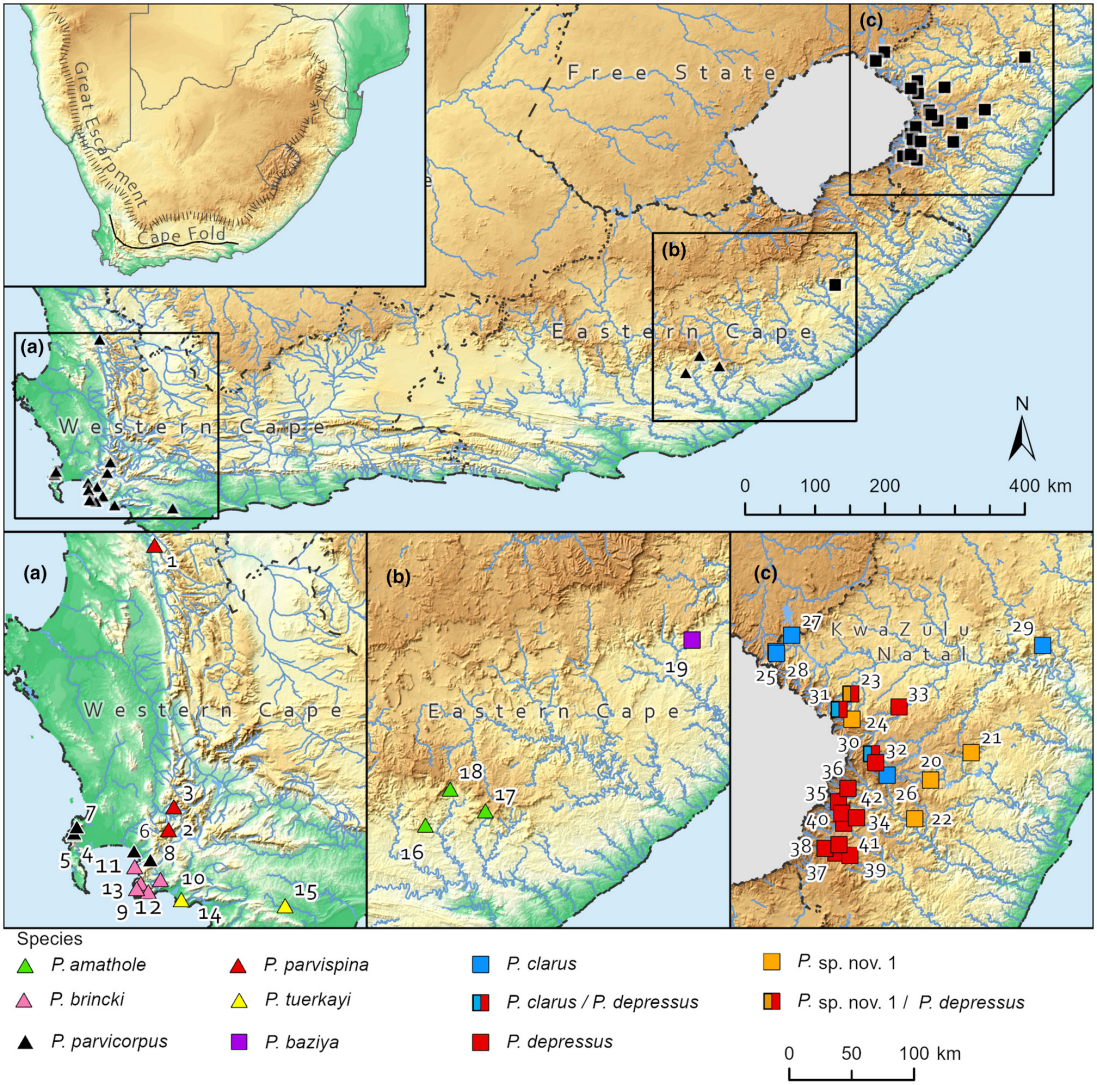
A map of South Africa showing all nine species of mountain freshwater crabs (*Potamonautes*) sampled during the present study. The numbers of the sample localities correspond to Table 1. All the Cape Fold Mountain species (clade A, map inserts A and B) are shown with a triangle, while all the Drakensberg Mountain (Great Escarpment) species (clade B, map inserts B and C) are shown with a square.

All described, plus one as yet, undescribed mountain living freshwater crab species present along the GE were included in the present study (Figure 1; Table 1). Upon capture, crabs were transported alive to the field laboratory and killed by freezing at −20°C for 24 h prior to being preserved in 96% ethanol. Sample sizes (*N*) ranged from one specimen to a maximum of five specimens per locality. Pereopods were taken from representative samples and the muscle tissue was used in DNA extraction. A handheld global positioning system (Garmin‐Trek Summit) was used to collect coordinates. Tissue samples from three *Potamonautes parvispina* sample localities (Keurbos, Berg River, and Wemmershoek Dam) were available from an earlier allozyme study (Daniels et al., 1998) and sequenced for both mtDNA loci. In addition, the three localities (Hogsback, Katberg, and Fort Fordyce Nature Reserve) of *P. amathole* were sequenced for both mtDNA (16S rRNA and the cytochrome oxidase subunit 1(COI), loci). For the three central CFM species (*P. brincki*, *P. parvicorpus*, and *P. tuerkayi*) the 12 localities for which COI data was generated by Wood and Daniels (2016) was sequenced for the 16S rRNA and combined with the COI data. During the present study, *P. baziya* specimens from the Baziya forest complex, Eastern Cape province were sequenced for both mtDNA markers. Along the Drakensberg Mountains (Great Escarpment, GE), freshwater crab specimens were collected at four new localities: Quadeni forest, Dargle forest, Karkloof Nature Reserve, and Impendle – with the latter three localities representing a new as yet, undescribed species, *Potamonautes* sp. nov. 1 (Figure 1). The additional Drakensberg Mountain localities were combined with mtDNA sequence data generated by Phiri and Daniels (2016). The nuDNA sequence data for the Drakensberg Mountain clade was obtained from Phiri and Daniels (2016).

**TABLE 1:** List of localities where the nine mountain‐stream freshwater crab species (*Potamonautes*) were sampled during the present study.

Locality #	Locality name	Mountain	Province	Species	*N*	S	E	Altitude (m)
1	Keurbos	CFM	Western Cape	*P. parvispina*	4	32 15.520	18 58.050	518
2	Berg River	CFM	Western Cape	*P. parvispina*	4	33 58.369	19 04.067	551
3	Wemmershoek dam	CFM	Western Cape	*P. parvispina*	4	33 50.170	19 06.340	310
4	Kirstenbosch	CFM	Western Cape	*P. parvicorpus*	3	34 00.054	18 23.298	765
5	Orange Kloof	CFM	Western Cape	*P. parvicorpus*	5	33 59.590	18 23.311	454
6	Blinkwater	CFM	Western Cape	*P. parvicorpus*	4	34 06.011	18 49.169	682
7	Platteklip	CFM	Western Cape	*P. parvicorpus*	5	33 57.344	18 24.470	512
8	Sir Lowry's Pass	CFM	Western Cape	*P. parvicorpus*	5	34 09.156	18 56.235	368
9	Rooiels	CFM	Western Cape	*P. brincki*	3	34 17.563	18 52.025	70
10	Palmiet River	CFM	Western Cape	*P. brincki*	5	34 16.213	19 00.575	192
11	Steenbras River	CFM	Western Cape	*P. brincki*	5	34 11.630	18 49.464	129
12	Betties Bay	CFM	Western Cape	*P. brincki*	1	34 20.529	18 55.329	205
13	Pringle Bay	CFM	Western Cape	*P. brincki*	5	34 19.428	18 50.298	112
14	Fernkloof NR	CFM	Western Cape	*P. tuerkayi*	5	34 23.370	19.16.351	120
15	Napier	CFM	Western Cape	*P. tuerkayi*	5	34 25.557	19 54.414	127
16	Fort Fordyce NR	CFM	Eastern Cape	*P. amathole*	4	32 41.232	26 29.945	957
17	Hogsback	CFM	Eastern Cape	*P. amathole*	3	32 36.026	26 55.852	1266
18	Katberg	CFM	Eastern Cape	*P. amathole*	4	32 28.132	26 40.653	1087
19	Baziya Forest Station	GE	Eastern Cape	*P. baziya*	4	31 33.917	28 25.176	999
20	Dargle Forest	GE	KwaZulu‐Natal	*P*. sp. nov. 1	3	29 29.042	30 03.090	1336
21	Karkloof NR	GE	KwaZulu‐Natal	*P*. sp. nov. 1	3	29 18.725	30 20.611	1177
22	Impendle	GE	KwaZulu‐Natal	*P*. sp. nov. 1	4	29 43.560	29 56.262	1188
23	Cathedral Peak NR	GE	KwaZulu‐Natal	*P*. sp. nov. 1/*P. depressus*	1/1	28 56.525	29 28.589	1814
24	Injasuthi NR	GE	KwaZulu‐Natal	*P*. sp. nov. 1	2	29 06.123	29 29.182	1451
25	Gudu Falls	GE	KwaZulu‐Natal	*P. clarus*	2	28 40.526	28 56.287	1952
26	Lothoni NR	GE	KwaZulu‐Natal	*P. clarus*	2	29 27.200	29 44.321	2260
27	Oliviershoek Pass	GE	KwaZulu‐Natal	*P. clarus*	2	28 34.329	29 03.132	1663
28	Mahai NR	GE	KwaZulu‐Natal	*P. clarus*	2	28 41.225	28 56.578	1503
29	Quadeni Forest	GE	KwaZulu‐Natal	*P. clarus*	1	28 38.195	30 51.513	1460
30	Highmoor NR	GE	KwaZulu‐Natal	*P. clarus*/*P. depressus*	1/3	29 19.157	29 37.519	1998
31	Monks Cowl NR	GE	KwaZulu‐Natal	*P. clarus*/*P. depressus*	2/2	29 02.385	29 23.561	1568
32	Kamberg NR	GE	KwaZulu‐Natal	*P. depressus*	2	29 22.518	29 39.380	1706
33	Lower Mzamkhulu	GE	KwaZulu‐Natal	*P. depressus*	2	29 01.492	29 49.523	1244
34	Garden Castle NR	GE	KwaZulu‐Natal	*P. depressus*	4	29 45.225	29 25.591	1819
35	Sani Pass	GE	KwaZulu‐Natal	*P. depressus*	4	29 37.212	29 23.341	2194
36	Vergelegen NR	GE	KwaZulu‐Natal	*P. depressus*	4	29 32.171	29 27.399	1596
37	Ndawana	GE	KwaZulu‐Natal	*P. depressus*	2	29 56.409	29 22.249	1655
38	Bushmans Nek	GE	KwaZulu‐Natal	*P. depressus*	2	29 54.690	29 17.477	2343
39	Coleford	GE	KwaZulu‐Natal	*P. depressus*	2	29 57.318	29 28.275	1516
40	Cobham	GE	KwaZulu‐Natal	*P. depressus*	2	29 41.462	29 24.709	1617
41	Rougham	GE	KwaZulu‐Natal	*P. depressus*	2	29 53.282	29 23.537	2337
42	Himeville	GE	KwaZulu‐Natal	*P. depressus*	2	29 43.201	29 31.163	1521

*Note*: The locality # numbers (1 to 42) correspond to those on the map (Figure 1) of South Africa. Mountain denotes the two types, CFM denoting the Cape Fold Mountains, and GE denoting the Great Escarpment (Drakensberg). Localities 4 to 15 were sequenced for the COI locus by Wood and Daniels (2016), while for localities 23–28, 30–42 both mitochondrial and nuclear loci data came from Phiri and Daniels (2016). Nature reserves are denoted by NR.

### 2.2 DNA extraction and PCR

DNA was extracted from the ethanol‐preserved pereopod muscle tissue of each specimen (Daniels et al., 2002) using a Nucleospin kit (Macherey‐Nagel), following the manufacturers protocol. Extracted DNA was stored at −20°C until required for PCR (Daniels et al., 2006). During the present study, two mitochondrial loci, the cytochrome c oxidase subunit I (COI), and the 16S rRNA locus were amplified using the primer pairs outlined in Daniels et al. (2002). These two markers have been extensively used in phylogenetic studies of Afrotropical freshwater crabs (Daniels et al., 2002, 2006, 2015; Phiri & Daniels, 2014, 2016; Wood & Daniels, 2016).

A 25 μL reaction solution for each sample was utilised for the PCR amplifications: Millipore water (14.9 μL), 25 mM MgCl_2_ (3.5 μL), 10 × Mg^2+^ free buffer (2.5 μL), 10 mM deoxyribonucleotide triphosphate solution (0.5 μL), 10 mM forward and reverse genetic marker primers (0.5 μL each), 0.1 U Taq polymerase (0.1 μL), and 2.5 μL of the 1:19 template DNA dilution. For the COI target marker, the PCR conditions were 94°C (4 min), [94°C (30 s), 42°C (40 s), 72°C (45 s)] for 36 cycles, and a final extension at 72°C (10 min). The same conditions were used for the 16S rRNA locus; however, the annealing temperature was 48°C. PCR products were electrophoresed (120 min. in a 1.5% agarose gel containing ethidium bromide) and the PCR products were then gel purified with the BioFlux purification kit (Bioer Technology Co., Ltd). Sequencing was performed at the Central Analytical Facility of the Stellenbosch University. DNA sequences were aligned and edited using muscle, as implemented in mega v. 7.0.18. To check for stop codons in the COI sequences were translated to amino acids using the online programme emboss‐transeq (http://www.ebi.ac.uk/emboss/transeq/); no stop codons were detected.

### 2.3 Phylogenetic reconstructions and divergence time estimation

We reconstructed a phylogeny of the mountain living *Potamonautes* using two species as outgroups (*P. ntendekaensis* and *P. sidneyi*) and using maximum likelihood (ML), and Bayesian inference (BI). The best‐fit partitioning scheme as well as the best substitution models for the respective partitions were found with partitionfinder v.2.1.1 using the Bayesian Information Criterion and considering GTR, TrN, HKY and JC models with and without gamma distributed substitution frequencies. This resulted in two partitions, one comprising the mitochondrial 16S rRNA gene (TrN + Γ), and one comprising the protein‐coding COI. Maximum likelihood analysis was conducted on the concatenated dataset in raxml v.7.2.7 (Stamatakis, 2006). The robustness of branches of the best ML tree was assessed with 1000 bootstrap replicates using the CAT algorithm for fast bootstrapping, while the final tree search was conducted under the GTR + Γ model for both partitions as less complex substitution models than GTR are not implemented in raxml. Uncorrected pairwise distances were calculated between sister species using the COI locus in PAUP v 4.0b10 (Swofford, 2002). Our data, based on sampling, were not suitable for population genetic analyses, hence we did not undertake an analyses of molecular variance.

The Bayesian phylogenetic reconstruction and divergence time estimation was executed in beast v. 2.4.7 (Bouckaert et al., 2014) by running the Marcov chain for 50 × 10^6^ iterations, sampling every 10,000 iterations (repeated once to check for convergence of both runs). Substitution models and data partitioning were implemented as suggested by partitionfinder (see above). Convergence of sampled parameters and potential autocorrelation was investigated in tracer v. 1.6 (Rambaut et al., 2014), ensuring an effective sample size for all parameters >100. We applied a Yule tree prior and an uncorrelated lognormal relaxed molecular clock after initial test runs (investigating if the standard deviation of the uncorrelated lognormal clock approaches zero), using potamonautid substitution rates (and their standard deviation) as priors that originate from a fossil calibrated phylogeny of the whole family Potamonautidae (Daniels et al., 2015): 0.81% per Ma for the rRNA locus (SD = 0.0013; linked clock models), 2.85% per Ma (SD = 0.005) for the COI locus. The maximum clade credibility tree was determined and annotated in treeannotator v. 2.4.1 (part of the beast package) after removal of 10% of the trees as burn‐in.

### 2.4 Biogeography

To infer the geographical origin of the mountainous freshwater crab lineages of southern Africa we conducted likelihood analyses of ancestral ranges based on a dispersal‐extinction‐cladogenesis model (DEC; Ree & Smith, 2008), including the possibility of founder‐event speciation (DECj; Matzke, 2012; for critical discussion of DECj see Ree & Sanmartín, 2018 and Matzke, 2022). We defined four regions: The South Cape region, the southern East Cape region, the northern East Cape region, and the KwaZulu‐Natal region. Thus, the coding of the areas follow the current knowledge of biogeographic distinct regions in the area (Clark et al., 2011a, 2011b). The maximum credibility tree of the divergence time estimation (pruned to single species tips and with outgroups removed) was used as phylogenetic information. Due to the very high posterior probabilities of recovered divergence events, we did not consider phylogenetic uncertainty in the biogeographical analysis. We allowed ancestral ranges to comprise a maximum of two areas, because we consider widespread ancestral ranges as extremely unlikely, given the known evidence for relatively fast geographical genetic differentiation followed by allopatric speciation in primary freshwater crabs (Jesse et al., 2010).

To test if either the DEC or DECj models of range evolution fit better to the data, and to further identify the most likely ancestral area, we additionally followed a model testing approach by conducting a set of analyses, restricting in each the possible dispersal direction. We set the dispersal parameter in the dispersal multiplier matrix to either 0 or 1 according to the following hypotheses: (1) origin in the south cape area and subsequent dispersal northwards; (2) origin in a (combined southern and northern) east cape area, (3) origin in the KwaZulu‐Natal region and subsequent dispersal to the southern areas. We calculated the three models as well as two unconstrained analyses both under the DEC and DECj models of range evolution and compared them based on the Akaike Information Criterion (AIC), ranking the models against the best model via ΔAIC. Biogeographical analyses were conducted with the R‐package BioGeoBEARS (Matzke, 2022).

### 2.5 Species delimitation using ASAP, bPTP, bGMYC, and STACEY

The candidate lineages for the species delimitation analyses were split into two groups. The first group comprised all specimens included in the study as per the phylogenetic analyses (hereafter referred to as total DNA), which were tested using both the 16S rRNA plus COI loci for all methods employed for both the CFM (A) and Drakensberg clades (B), respectively. The second group comprised only specimens sourced from the Drakensberg Mountains using the data generated by Phiri and Daniels (2016) (Table 1). All four delimitation methods were employed for both groups. The Drakensberg Mountain group (B) was analysed multiple times with each analysis making use of the separate set of loci. These included an initial analysis of the mtDNA only (COI, 16S rRNA, and 12S rRNA), a second dataset of the nuDNA only (28S, DecANT, and PEPCK), and a final dataset of all loci in combination (hereafter referred to as combined DNA; COI, 16S rRNA, 12S rRNA, 28S, DecANT, and PEPCK) (Phiri & Daniels, 2016). The newly generated Drakensberg Mountain specimens were not included in the fine‐scale analyses since we only had two mtDNA loci (16S rRNA and COI) and no nuDNA data.

The first method employed for the species delimitation comprised the newly developed assemble species by automatic partitioning (ASAP) (Puillandre et al., 2021). This method uses genetic distances to hierarchically cluster species partitions (https://bioinfo.mnhn.fr/abi/public/asap). ASAP first assigns a probability that each new clustering is a new species and then computes the relative width of the barcode gap of a partition in relation to the previous partitions. These metrics are combined into an ASAP score to rank all partitions detected in the analyses. Since ASAP is an exploratory method that does not consider the evolutionary history among sequences, we report the first two partitions ranked by ASAP score, using p‐distances and the default setting splitting groups below probability <.01.

A Bayesian implementation of the Poison tree processes (bPTP) was run on the online via the bPTP web server (https://species.h‐its.org/ptp/) for its ability to delimit species without *a priori* knowledge of population parameters (Zhang et al., 2013). A maximum likelihood tree for each dataset was used for the analysis which was run for 500,000 MCMC generations with a thinning value = 100 and burn‐in = 0.20. The outgroup taxa were defined and removed from the analysis to improve the delimitation results. The convergence of the MCMC chain was visually confirmed as recommended by Zhang et al. (2013).

Lastly, we also employed a Bayesian implementation of the GMYC model using the R package bGMYC (Reid & Carstens, 2012). All gene alignments were run in BEAST2 under a HKY nucleotide substitution model using a strict clock. To account for error in phylogenetic estimation, 500 post‐burn‐in trees were randomly selected for analysis. A Markov chain was run for 50,000 generations, sampling the chain every 1000th generation and 4000 generations were discarded as burn‐in. A uniform prior for the number of species was applied to each dataset with a lower bound of three and an upper bound of 139 (number of tips) for the total DNA, whereas all Drakensberg Mountain datasets (mtDNA, nuDNA, and combined DNA) comprised a lower bound of three and an upper bound of 52. Convergence was assessed visually by examining the performance of the chain. The ‘check rates’ function was used to determine the rate of branching of the coalescent model relative to that of the mixed Yule model.

Additionally, we employed a multilocus, coalescent model using (STACEY ver. 1.2.1) for its demonstrated effectiveness in species boundaries validation (Busschau et al., 2019; Jacobs et al., 2018; Klimov et al., 2019; Tomasello, 2018). Thus, species tree estimation and a species delimitation analysis were performed in STACEY (ver. 1.2.1, http://www. beast2.org/, Jones, 2017) in BEAST2, for each of the datasets. The estimated number of putative species in STACEY ranges from one to the number of putative clusters specified. Each locality was defined as a taxon set or minimal cluster, without *a priori* species definition. The input files (.xml) were created using BEAUti, implementing a mixed Yule model before estimating the species tree using the following prior settings: Collapse Height = 0.0001; Collapse Weight = 0.5 using a beta prior (1.1) around (0.1); bdcGrowthRate = log normal (M = 4.6, S = 1.5); pop‐PriorScale = log normal (M = 7, S = 2); relativeDeathRate = beta (alpha = 1.0, beta = 1.0); and a strict model to describe the molecular clock. For the total DNA ploidy levels, the COI locus was set to 0.5, and the 16S rRNA locus was set to 2.0. For the mtDNA analysis, ploidy levels were set to 2.0 for the 12S rRNA and 16S rRNA loci and 0.5 for the COI locus; the nuDNA ploidy levels were all set to 2.0, and the combined DNA dataset comprised ploidy level equal to each of the aforementioned values. The use of equal ploidy settings for all loci represents a more robust approach by avoiding the disproportionate influence of mitochondrial partial‐sequence data and the overestimation of the number of putative species (Vitecek et al., 2017). Equal ploidy levels were attempted for the mtDNA (COI) data but was deemed too conservative. The MCMC analysis was run for 100,000,000 generations, saving the result every 5000 generations. The obtained log files were analysed with Tracer to verify convergence (ESS > 200) of the analysis, and SpeciesDelimitationAnalyser (http://indriid.com/software.html; Jones et al., 2015) was used to process the log files and to examine the clusters of species assignments. Posterior probabilities of localities belonging to the same cluster were visualised in a similarity matrix constructed in R Studio without requirement of a specific package. A second analysis was performed in STACEY, in which the clusters visualised in the similarity matrix were defined as minimal clusters.

### 2.6 Morphometric analyses

Von Rintelen et al. (2010) employed a discriminant functions analyses on their morphometric data and demonstrated the presence of adaptive radiations in freshwater shrimp of *Caridina* from the Indonesian island of Sulawesi. We used a similar approach. For four of the freshwater crab species (*P. amathola*, *P. baziya*, *P. tuerkayi*, and *Potamonautes* sp. nov. 1), we had very low sample sizes (on average < 30 specimens), thus we excluded these species from the analyses. Five freshwater crab species were included, *P. brincki*, *P. parvicorpus*, and *P. parvispina* represented the CFM clade, while *P. clarus* and *P. depressus* represented the Drakensberg clade, for a total of for 816 specimens. A digital calliper was used to measure the following seven carapace variables: carapace length (CL); the carapace width at widest point (CWW); the width of the posterior margin of the carapace (CWP); the distance between the postfrontal crest and the anterior margin of the carapace (PFCD); the frontal width, measured between the medial margins of the orbits (FW); the distance between the exorbital teeth (CWA); and the carapace height (CH). All measurements are given in millimetres (mm). The variables were log transformed and the data was subjected to a discriminant functions analyses in SPSSv29.0.

### 2.7 Climatic niche comparison

Differences in climate between the western (Cape Fold Mountains, including the following species, *P. brincki*, *P. parvicorpus*, and *P. parvispina*) and eastern (Drakensberg Mountains, GE, including the following two species, *P. clarus* and *P. depressus*) freshwater crab taxon groups were statistically assessed. The four remaining species (*P. amathole*, *P. baziya*, *P. tuerkayi*, and *Potamonautes* sp. nov. 1) are narrow endemics, for which we had less than five distribution records and these were thus excluded from further analyses. All 19 bioclimatic variables were downloaded from the WorldClim database (Hijmans et al., 2005) at a resolution of 30 s. Climatic data was extracted for each locality using the R‐package Raster (Hijmans, 2022) in RStudio 2022.07.1 (RStudio Team, 2020), with duplicate or nearby localities which shared the same raster cell removed from the analyses using the R package spThin (Aiello‐Lammens et al., 2015). The variables were all standardised for the multivariate analysis, and the relative climates of each population were analysed using a principal component analysis (PCA) in with the R function pcrcomp, after which the results were visualised using the R package ggplot2 (Wickham, 2016). A permutational multivariate analysis of variance (PERMANOVA) with 9999 permutations was used to statistically test the differences in climate among the two populations using the r package vegan (Oksanen et al., 2022), and a Kruskal–Wallis test was conducted on each variable in order to identify the significantly different variables which contributed to the overall variation. Finally, Spearman rank correlation tests were conducted in order to determine whether the variation in each climatic variable was correlated with latitude and longitude.

### 2.8 Climatic nice modelling

Species distribution models (SDMs) were constructed for the two *Potamonautes* taxon groups (clades A and B) in order to visualise the differences in their ecological niches. All 19 bioclimatic variables used in the previous analyses were cropped to the geographical extent of South Africa using QGIS 3.24.2 (QGIS.org, 2022). Correlated variables (with a Pearson's correlation coefficient > 0.7) for the entire dataset were assessed using the R package CorrPlot (Wei & Simko, 2021). Additionally, the R package usdm (Naimi et al., 2014) was used to conduct a variance inflation factor (VIF) analysis using a threshold of five, with the remaining limiting climatic variables used to generate the models. Subsequently, an additional, categorical variable was used which consisted of a binary raster indicating the geographical presence or absence of South African river systems, mapped to the same resolution as the WorldClim bioclimatic variables. A custom R script was used to specify a fixed distance buffer of 100 km based on the occurrence records for model building in order to correct the models for overprediction, as well as to produce minimum convex polygons of the distribution for each taxon. The R package ENMeval 2.0 (Kass et al., 2021) was used to determine the optimum feature class and regularisation multiplier for model construction and to convert the continuous probability surfaces to binary models representing either suitable or unsuitable climatic conditions based on the maximum test specificity and sensitivity thresholds. Maxent 3.4.1 (Phillips et al., 2006, 2017) was used to determine the permutation importance (PI) of each variable to each model. QGIS was used to export the final map.

## 3 RESULTS

### 3.1 Phylogenetic analyses and divergence time estimation based on the combined mtDNA

**FIGURE 2:**
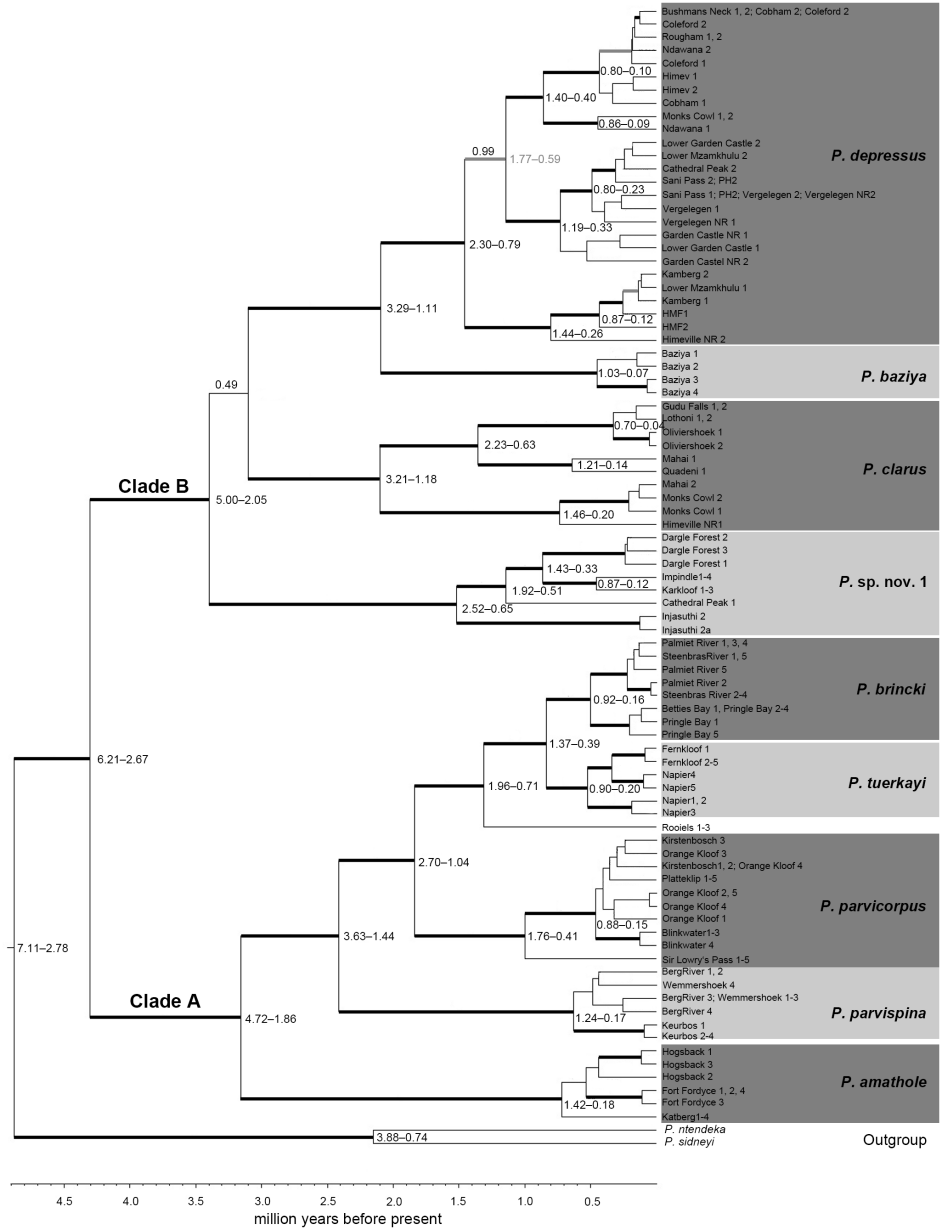
A dated Bayesian inference tree topology derived from the combined mtDNA sequence data (16S rRNA + COI) showing two distinct mountain freshwater crab (*Potamonautes*) clades. Clade A represents all five of the Cape Fold Mountain (CFM), while clade B represents all four of the Drakensberg Mountain (GE) freshwater crab species. Posterior probability values >.95 (p*P*) together with bootstrap values >75% are shown above and below branches, respectively.

The combined mtDNA dataset (16S rRNA + COI) comprised a 1434 bp fragment. All novel generated 16S rRNA and COI sequences were deposited in GenBank (for the 89 specimens sequenced for the 16S rRNA locus the accession numbers are OQ789108‐OQ789196; for the 38 specimens sequenced for the COI locus the accession numbers are OQ788447–OQ788484). The BI and ML tree topologies were near identical hence only the BI topology is shown (Figure 2). The monophyly of the South African mountain living crabs was observed and two distinct clades were retrieved. These two sister clades shared a most recent common ancestor (MRCA) 4.31 Mya [95% HPD: 2.67–6.21 Mya]. Clade A comprised all the species from the CFM, with *P. amathole* (Hogsback, Katberg, and Fort Fordyce) situated basally to the remainder of the four endemic CFM freshwater crab species. Within the latter clade, *P. parvispina* (Cederberg, and Kouebokkeveld Mountains) was sister to *P. parvicorpus* (Table Mountain, Helderberg, and Jonkershoek Mountains). The latter species was sister to *P. tuerkayi* (Overberg Mountains) and *P. brincki* (Hottentots Holland Mountains). Within clade A, the species shared a MRCA 3.16 Mya [95% HPD: 1.86–4.72 Mya], within the Western Cape Fold mountains, the Cederberg lineage, *P. parvispina* diverged from the central Western Cape lineages 2.41 Mya [95% HPD: 1.44–3.63 Mya]. Within the central CFM clade, *P. parvicorpus* diverged 1.87 Mya [95% HPD 2.70–1.04 Mya] from *P. tuerkayi* and *P. brincki*, while the latter two species diverged 1.33 Mya [95% HPD: 1.96–0.71 Mya]. Clade B comprised species exclusive to the GE, including the Drakensberg Mountains. Divergence time estimation suggested that species in this clade shared a MRCA 3.40 Mya [95% HPD: 2.05–5.00 Mya]. *Potamonautes* sp. nov. 1 from Dargle, Karkloof, Injasuthi, and Cathedral Peak was basal in this clade. In addition, within the latter clade *P. clarus* from Monks Cowl NR, Mahai, Himeville, Quadeni, Oliviershoek Pass, Lothoni, and Gudu Falls diverged 2.19 Mya [95% HPD: 1.18–3.21 Mya] from its sister clade comprising *P. baziya* sister to *P. depressus* (from Himeville, Kamberg, Vergelegen, Sani Pass, Cathedral Peak, Monks Cowl, Coleford, and Cobham). The latter two species diverged 2.20 Mya [95% HPD: 1.11–3.29 Mya].

In the CFM (Clade A), the uncorrected ‘p’ distance using the COI locus ranged between 8.00% *P. amathole* and *P. parvispina*, while between *P. brincki* and *P. parvicorpus* it was 8.38%. In the Drakensberg Mountain (Clade B), sister species pairs the uncorrected distance between *Potamonautes* sp. nov. 1 and *P. depressus* was 8.00%, while between *Potamonautes* sp. nov. 1 and *P. clarus* it was 8.32%.

### 3.2 Biogeography

**FIGURE 3:**
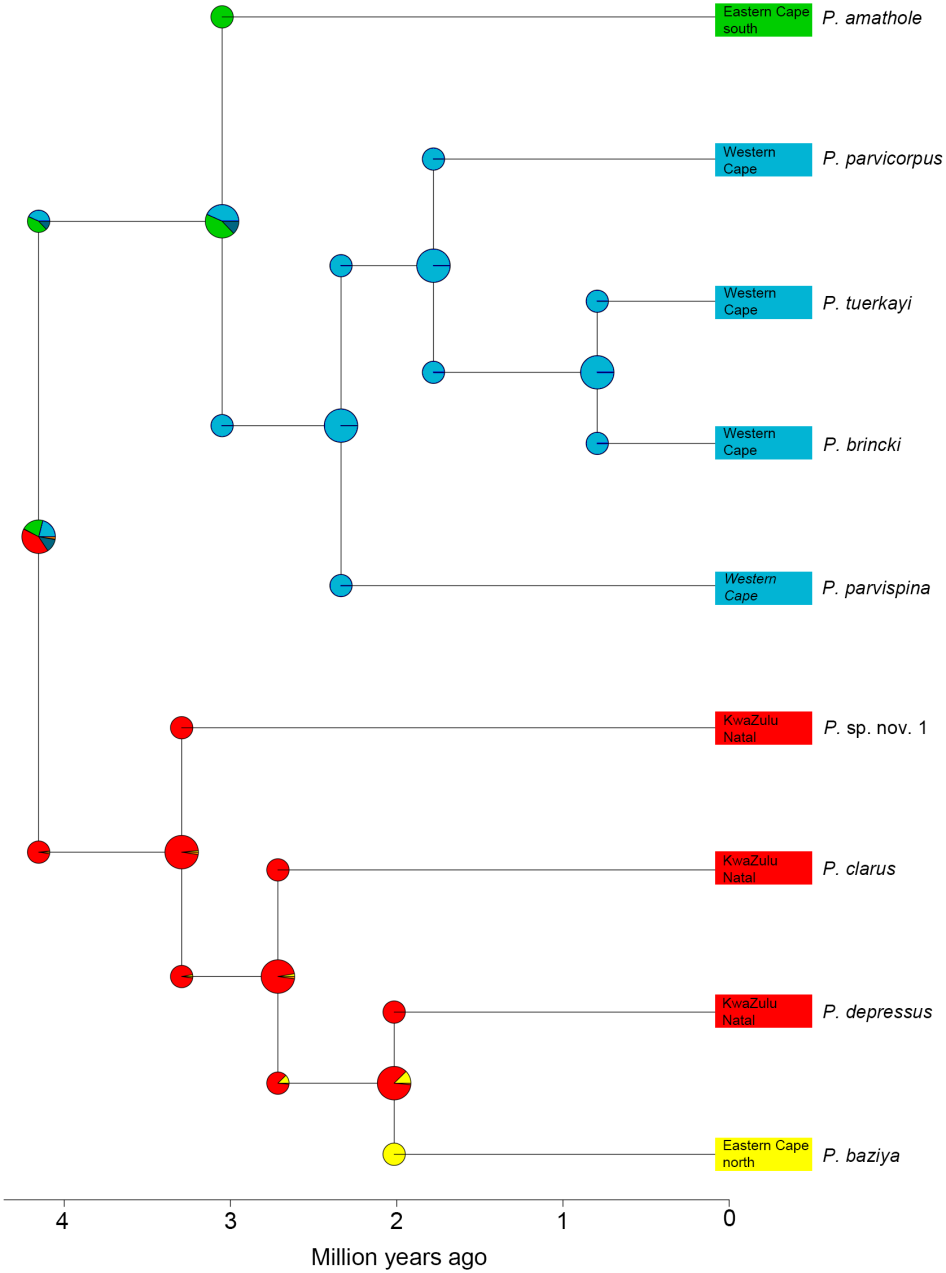
Result of the biogeographical inference based on the unconstrained DECj model of range evolution. The boxes represent the estimated ranges. In cases where ranges covered several areas the boxes contain several letters. The pie charts represent the relative probabilities for occurrence among each of the provinces included.

The DECj model of range evolution that additionally considers founder‐event speciation fitted better to the data than the plain DEC model (ΔAIC = 14.43). Consequently, we only discuss the results of the biogeographical analysis based on DECj. The obvious nesting of *P. baziya* of the northern Eastern Cape region within the Drakensberg Mountains (clade B) indicates a recent dispersal southward corroborated by the biogeographical analysis (Figure 3). In contrast, the only species of the southern Eastern Cape region (*P*. *amathole*) is sister to the CFM clade (A), and biogeographical estimation seems equivocal for the common ancestor of both clades (best estimate: southern east Cape region). Consequently, the range of the most recent common ancestor of the mountain freshwater crab clade remains ambiguous, with an ancestral origin in the Drakensberg Mountains (clade B) region being the best estimate. The latter northern‐origin biogeographical hypothesis received strong support by the model testing approach, with a potential Eastern Cape origin being slightly less supported (Table 2). Given the present phylogenetic and biogeographical data, we consider an origin of the South African mountain river crabs in the northern part of its range with subsequent dispersal southwards as the most likely scenario.

**TABLE 2:** Results of the biogeographical model testing with the models ranked according to their delta AIC relative to the best model.

Model	d	e	j	k	LnL	AIC	ΔAIC
DECj Drakensberg origin	0.00	0.00	0.30	9	−8.12	34.24	—
DECj eastern cape origin	0.00	0.00	1.72	10	−7.96	35.91	1.7
DECj south cape origin	0.00	0.00	0.38	9	−12.01	42.03	7.8
DEC eastern cape origin	0.18	0.00	—	10	−14.41	48.82	14.6
DECj unconstrained	0.00	0.00	0.10	16	−8.99	49.98	15.7
DEC Drakensberg origin	0.26	0.12	—	9	−16.48	50.95	16.7
DEC south cape origin	0.35	0.16	—	9	−18.46	54.91	20.7
DEC unconstrained	0.07	0	—	16	−16.21	64.41	30.2

Abbreviations: d, dispersal/range expansion rate; e, extinction/range contraction rate; j, jump dispersal rate/no ancestral areas inherited; k, free parameters in the dispersal multiplier matrix.

### 3.3 Species delimitation using ASAP, bPTP, bGMYC, and STACEY

**FIGURE 4:**
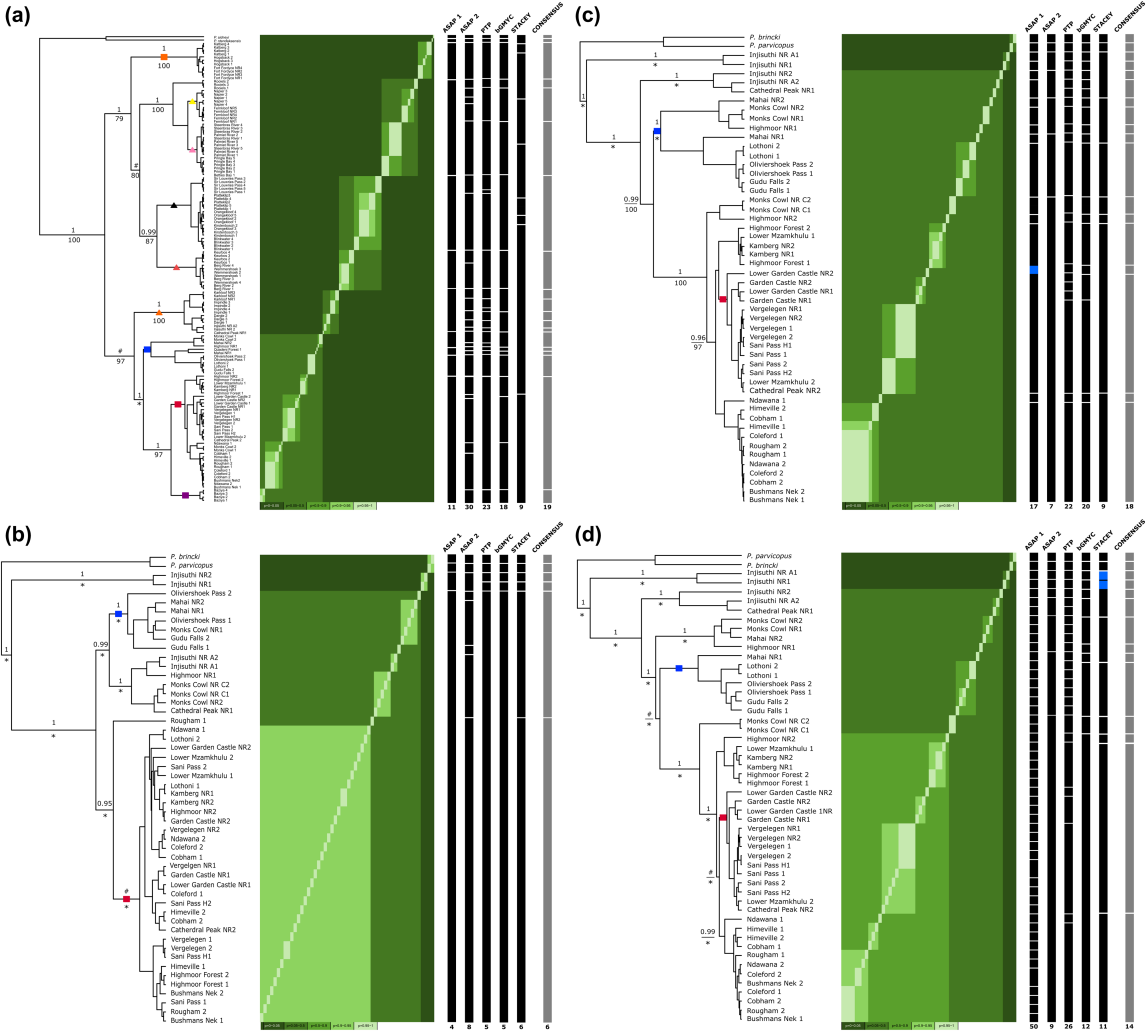
Species delimitation result summary, for all *Potamonautes* specimens and for the Great Escarpment (Drakensberg Mountains) (clade B) specific. (a) Represents the species delimitation results of the combined mtDNA (16S rRNA and COI) sequence dataset for all the mountain living freshwater crab species. (b) The species delimitation results focussing exclusively on the nuDNA sequence data (28S, DecANT, and PEPCK) for the Drakensberg Mountains clade (clade B). (c) The species delimitation results focussing exclusively on the mtDNA (12S rRNA, 16S rRNA, and COI) for the Drakensberg Mountain clade (clade B) only, and finally (d) represents the combined mt + nuDNA sequence data for the latter clade (clade B). All trees represent an ultrametric BEAST tree with the matrix representing the bGMYC results, support is shown in the key below each matrix. The five black coloured vertical bars to the right of each matrix represent alternative taxonomies with each segments representing distinct species according to the respective delimitation method employed. Blue segments within the latter bars represent the retrieval of individual species as putative species by their respective method. Statistical support for nodes is derived from the species tree methods bGMYC and STACEY (above branch, posterior probability values derived from the BEAST analysis) and bPTP (below branch as bootstrap values derived from the maximum likelihood analysis). Non‐supported nodal relationships (<.95 p*P*/<75%) are shown by their relevant symbols (#) bGMYC and STACEY, and (*) bPTP. The same symbols used on the map (Figure 1) is also present for each species on the species delimitations topologies.

The species delimitation results exhibited limited overlap between analyses conducted on all specimens included in the present study (Figure 4a), albeit with the number of putative species retrieved falling within a similar range. The first two partitions retrieved by ASAP differed grossly in the amount of putative species identified, with the first and second partitions retrieving nine and 28 putative species, respectively. The bPTP analysis retrieved a similar result to partition two of the ASAP analysis with 21 putative species identified. Among both the former results, there was clear overestimation of species when compared to the phylogenetic results which can be attributed to high intraspecific diversity where specimens were sourced from the same localities, resulting in over‐splitting such as, for example, at the Sir Lowry's Pass and Impendle localities. The bGMYC analysis retrieved five putative species for the total mtDNA dataset (15 at *p* > .95; 34 at *p* > .95; 41 at *p* > 1), retrieving Injasuthi 1 and Injasuthi 1A as a distinct group falling basal to the remainder of the topology. When defining localities as minimum clusters, the multilocus species delimitation analysis with STACEY performed on all study specimens retrieved 27 putative species. In several instances, single localities were retrieved as putative species, a relatively uncommon result when incorporating mitochondrial gene fragments, but likely due to geographic isolation over the broad sampling area (Figure 4a).

The results for each of the Drakensberg Mountain datasets (clade B) exhibited improved clarity as to the number of putative species retrieved with overlapping results. As expected, the varied mutation rates between the various loci were reflected by the results from the nuDNA dataset (Figure 4b) yielding the lowest number of putative species across all analyses, while the mtDNA (Figure 4c) and combined DNA datasets (Figure 4d) interchanged in retrieving the highest number of putative species per analysis, with the latter retrieving a relative median between the mtDNA and nuDNA datasets most often. For the mtDNA dataset, the first two partitions of the ASAP analysis retrieved 15 and five putative species, respectively. The ASAP results for the nuDNA dataset retrieved one and six putative species (Figure 4b), respectively, while the combined DNA dataset had 50 and 10 putative species retrieved by ASAP (Figure 4d). The bPTP analysis followed a similar trend, retrieving 20 putative species for the mtDNA dataset, two putative species for the nuDNA dataset, and 26 putative species for the combined DNA dataset.

Interestingly, at a threshold of *p* > .5, the bGMYC analysis retrieved two putative species for all Drakensberg datasets, identifying the same two groups as distinct (mtDNA = 18 at *p* > .95; 24 at *p* > .95; 27 at *p* > 1; nuDNA = 8 at *p* > .95; 48 at *p* > .95; 50 at *p* > 1; combined DNA = 10 at *p* > .95; 24 at *p* > .95; 39 at *p* > 1). The first comprised the two specimens from the sample site Injasuthi 1 and Injasuthi 1a which fell basal relative to the remainder of the topology, constituting the second group. The distinct retrieval of the two Injasuthi specimens was congruent with the bGMYC results of the total DNA analysis, the ASAP results of the mtDNA and combined DNA datasets, and with the STACEY results of the mtDNA analysis. However, these specimens were retrieved as seperate putative species or rather Injasuthi 1 and Injasuthi 2 were retrieved as individual putative species with an identical topological placement (bPTP; mtDNA and combined DNA; Figure 4c,d). These results warrant further investigation at Injasuthi and suggest that the sample site Injasuthi 1 may harbour a novel species. The STACEY results were corroborative of the those from the other methods, providing improved species resolution and boundaries in addition. The STACEY result for the mtDNA dataset retrieved eight putative species with distinct boundaries, apart from a partial overlap with the non‐independent clustering of species' 5 and 6 comprising Cathedral Peak, Highmoor Forest, Kamberg, Lower Garden Castle, and Lower Mzamkhulu and Vergelegen NR, Sani Pass, and Garden Castle NR, respectively (Figure 4b–d). The STACEY result for the nuDNA was congruent with the results of the bPTP and bGMYC (*p* > .5) analyses retrieving two putative species, once again indicative of the conservative mutation rate of these markers. Lastly, for the combined DNA dataset, STACEY also retrieved eight putative species, however, with different species clusters when compared to the mtDNA dataset due to the inclusion of nuclear markers.

### 3.4 Morphometric analyses

**FIGURE 5:**
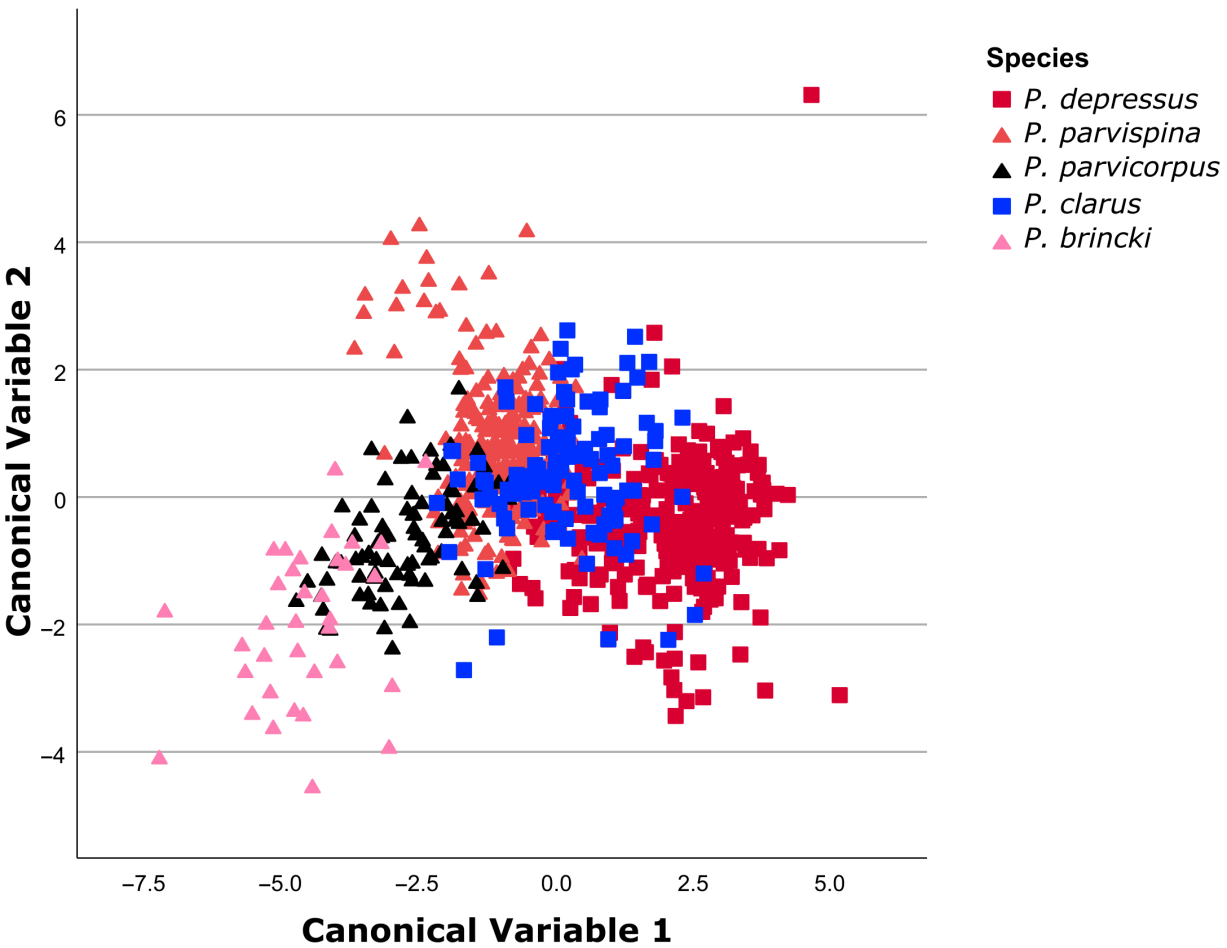
A discriminant functions analysis (DFA) of the log transformed carapace variables for the five mountain‐dwelling freshwater crab (*Potamonautes*) species.

The first two canonical variables contributed 88.1% to the total variation between the five freshwater crab species (Figure 5). A two‐dimensional plot of the individual scores along the first and second canonical variables based on the logarithmically transformed carapace variables for the five freshwater crab species showed a moderate degree of differentiation. The classification functions for the five species are presented in Table 3 and provides support to their genetic distinctiveness.

**TABLE 3:** Percentage correct a posteriori classification to the groups based on the morphometric classification function of the carapace variables for five mounting‐dwelling *Potamonautes* species.

Species	Species					Correct classification (%)
	*P. depressus*	*P. parvispina*	*P. parvicorpus*	*P. clarus*	*P. brincki*	
*P. depressus*	265	19	0	36	0	82.8
*P. parvispina*	2	241	7	9	0	93.1
*P. parvicorpus*	0	13	65	7	1	75.6
*P. clarus*	14	25	3	73	0	63.5
*P. brincki*	0	2	6	0	27	77.1

### 3.5 Climatic niche comparison

**FIGURE 6:**
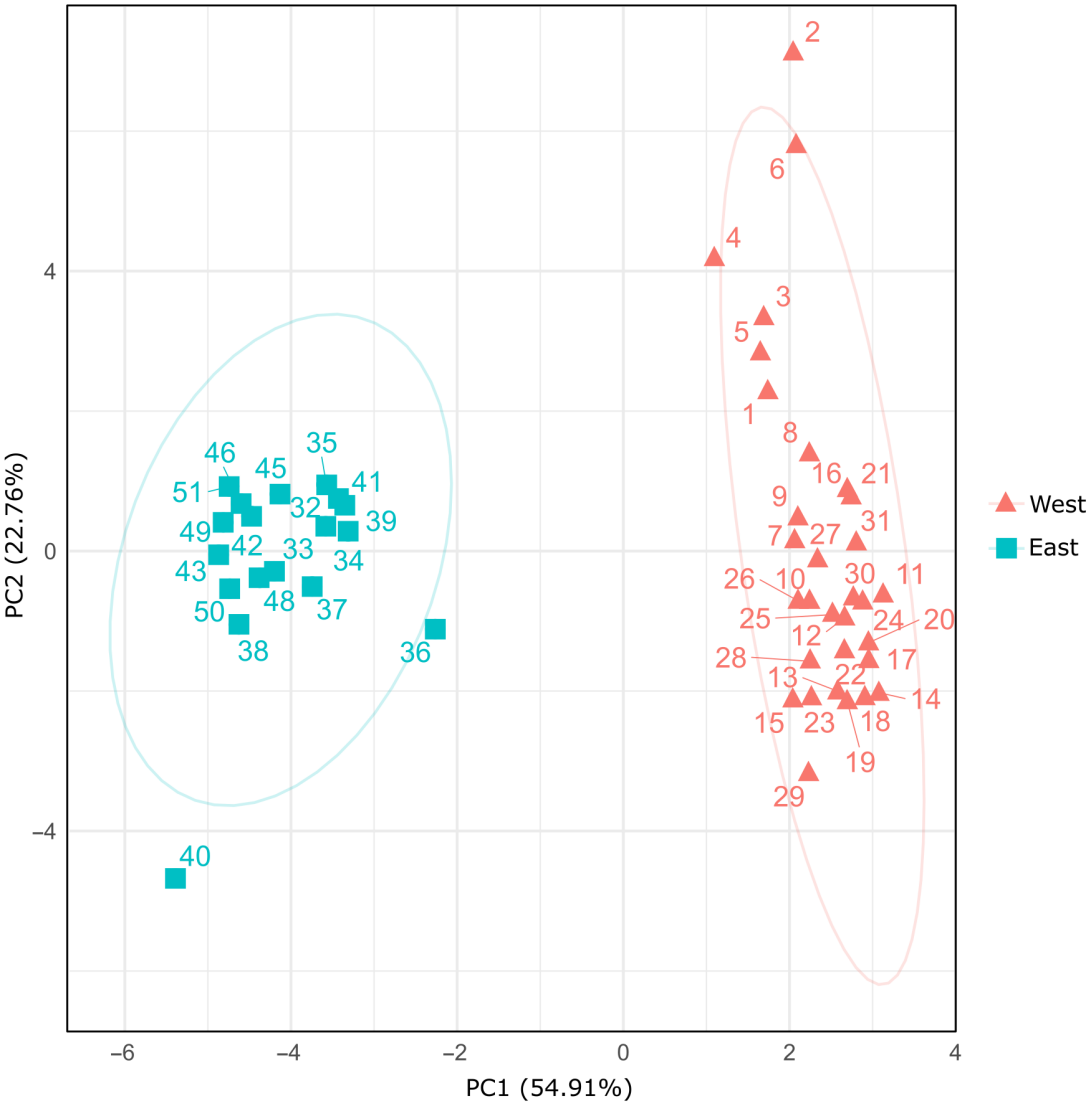
Principal components analysis on the bioclimatic variables at the sampling localities of the western (Cape Fold Mountains, Clade A) and eastern (Drakensberg Mountain, Clade B) group of freshwater crabs.

The PCA on the bioclimatic variables at the sampling localities (Figure 6) showed significant differences between the western (CFM species in Clade A) and eastern (Drakensberg Mountains, GE species, Clade B) *Potamonautes* groups, with the western group displaying greater levels of climatic heterogeneity within its distribution. The first two principal components explained 77.67% of the total variance (PC1 – 54.91%; PC2 – 22.76%), with the PC loadings (Table 4) showing that the variable ‘precipitation of the warmest quarter’ (Bio18) had the highest loading value within the first principal component, and that both ‘temperature seasonality’ and ‘maximum temperature of the warmest month’ (Bio4 and Bio5, respectively) showed the highest loading values within the second principal component. Both the PERMANOVA results (Table 2) and the Kruskal–Wallis tests (Table 5) demonstrated that the bioclimatic variables were significantly different between the two populations. All of the bioclimatic variables also demonstrated a significant correlation with both latitude and longitude (Spearman's correlation; Table 6A,B).

**TABLE 4:** List of bioclimatic variables used in the principal components analyses between the two freshwater crab clades (*Potamonautes*) from the Cape Fold Mountains (Clade A) and the Drakensberg Mountains (Clade B).

Variable	Environmental parameter	Principal component 1	Principal component 2
bio_1	Annual mean temperature	0.18695234	0.208610458
bio_2	Mean diurnal range	−0.231634972	0.287365331
bio_3	Isothermality	−0.248275669	−0.015613337
bio_4	Temperature seasonality	−0.117058591	0.386838471
bio_5	Max temp of warmest month	0.131857571	0.389820166
bio_6	Min temp of coldest month	0.28831773	−0.039446009
bio_7	Temperature annual range	−0.197696163	0.327524988
bio_8	Mean temp of wettest quarter	−0.243129904	0.023536877
bio_9	Mean temp of driest quarter	0.295592763	0.120340865
bio_10	Mean temp of warmest quarter	0.169888135	0.310391908
bio_11	Mean temp of coldest quarter	0.235022176	0.055968493
bio_12	Annual precipitation	−0.205356558	−0.267280676
bio_13	Precipitation of wettest month	−0.238148068	−0.189903395
bio_14	Precipitation of driest month	0.222115968	−0.270484127
bio_15	Precipitation seasonality	−0.250265077	0.021027205
bio_16	Precipitation of wettest quarter	−0.227441255	−0.215646567
bio_17	Precipitation of driest quarter	0.188254529	−0.312035374
bio_18	Precipitation of warmest quarter	−0.303373246	−0.044489253
bio_19	Precipitation of coldest quarter	0.275336254	−0.147513741

**TABLE 5:** Kruskal–Wallis test results for the environmental parameters tested.

		H (χ^2^)	*p* (same)	A (*N* = 31)	B (*N* = 18)
Bio1	Annual mean temperature	19.06	1.27E‐05	15.87258	13.75116
Bio2	Mean diurnal range	21.585	3.38E‐06	10.03387	13.04676
Bio3	Isothermality	31.355	2.15E‐08	52.11519	55.87252
Bio4	Temperature seasonality	5.0168	0.0251	336.8616	367.7904
Bio5	Max temp of warmest month	10.689	0.001078	26.01935	23.60556
Bio6	Min temp of coldest month	32.306	1.32E‐08	6.7580645	0.2444444
Bio7	Temperature annual range	14.255	0.0001596	19.26129	23.36111
Bio8	Mean temp of wettest quarter	27.1	1.93E‐07	11.76183	17.68889
Bio9	Mean temp of driest quarter	33.483	7.19E‐09	19.848925	9.026852
Bio10	Mean temp of warmest quarter	21.203	4.13E‐06	19.91882	17.72778
Bio11	Mean temp of coldest quarter	24.161	8.86E‐07	11.761828	8.695371
Bio12	Annual precipitation	30.665	3.07E‐08	646.3226	947.7778
Bio13	Precipitation of wettest month	32.667	1.09E‐08	101.0323	164.8889
Bio14	Precipitation of driest month	23.482	1.26E‐06	19.77419	10.38889
Bio15	Precipitation seasonality	31.588	1.91E‐08	57.98141	73.85566
Bio16	Precipitation of wettest quarter	32.539	1.17E‐08	289.5161	459.2778
Bio17	Precipitation of driest quarter	17.419	3.00E‐05	64.09677	43.61111
Bio18	Precipitation of warmest quarter	33.521	7.05E‐09	65.25806	459.05556
Bio19	Precipitation of coldest quarter	33.533	7.01E‐09	289.51613	46.38889

**TABLE 6:** Spearman correlation with latitude and longitude.

BioClimVar	ρ	*p* value	ρ	*p* value
bio_1	−0.588295322	8.77E‐06	−0.5941119	6.78E‐06
bio_2	0.752933977	4.36E‐10	0.8329421	1.15E‐13
bio_3	0.613061224	4.69E‐06	0.6683673	3.96E‐07
bio_4	0.559285714	4.03E‐05	0.612449	4.81E‐06
bio_5	−0.221989676	0.125273117	−0.1485038	3.09E‐01
bio_6	−0.821464133	4.79E‐13	−0.8823455	5.37E‐17
bio_7	0.683712293	6.14E‐08	0.7652148	1.53E‐10
bio_8	0.502576663	0.000233288	0.5110975	1.75E‐04
bio_9	−0.637277413	8.49E‐07	−0.6545742	3.36E‐07
bio_10	−0.504860318	0.000216128	−0.4699069	6.58E‐04
bio_11	−0.709730089	1.13E‐08	−0.7624369	1.95E‐10
bio_12	0.515638554	0.000149591	0.7144752	8.15E‐09
bio_13	0.540485395	6.10E‐05	0.7256999	3.65E‐09
bio_14	−0.818140663	7.10E‐13	−0.6104265	3.21E‐06
bio_15	0.637959184	1.57E‐06	0.5533673	5.01E‐05
bio_16	0.540759838	6.04E‐05	0.7334473	2.05E‐09
bio_17	−0.748519157	6.27E‐10	−0.5770335	1.42E‐05
bio_18	0.51756187	0.000139902	0.706402	1.42E‐08
bio_19	−0.809773288	1.85E‐12	−0.6654244	1.83E‐07

*Note*: The first set of values are for western Cape Fold Mountain group (Clade A), while the second set of values are for the eastern Drakensberg Mountain group (Clade B).

### 3.6 Climatic niche modelling

**FIGURE 7:**
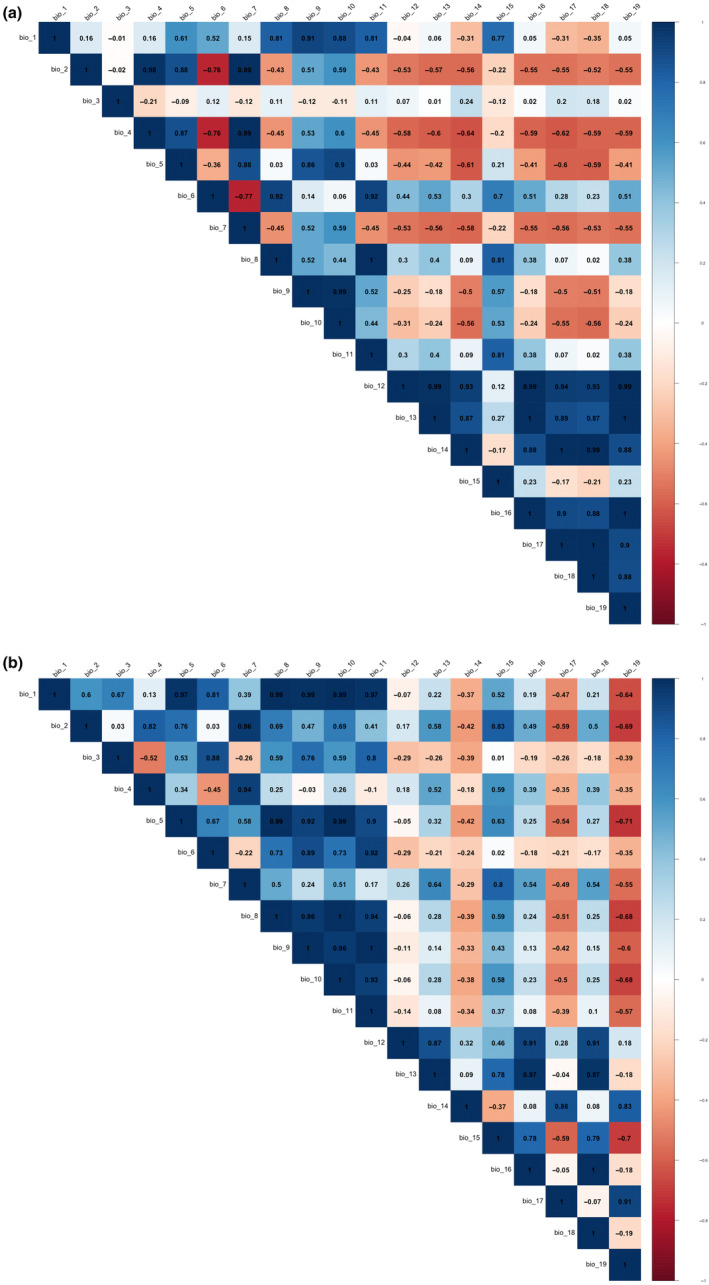
(a, b) Correlations of the bioclimatic variables at the sample localities. (a) representing the western group (Clade A – Cape Fold Mountains) and (b) representing the eastern group (Clade B – Drakensberg Mountains).

The bioclimatic variable correlation analyses (Figure 7) resulted in different sets of variables being retained for model construction for each taxon group. For the western group, five variables were retained (Bio1, Bio3, Bio4, Bio13, and Bio15), while six variables were retained for the eastern group (Bio3, Bio4, Bio6, Bio12, Bio14, and Bio16). Both SDMs performed well, with consistently high AUC_TEST_ and AUC_TRAIN_ scores, as well as low OR10 scores, indicating good model discrimination ability.

**FIGURE 8:**
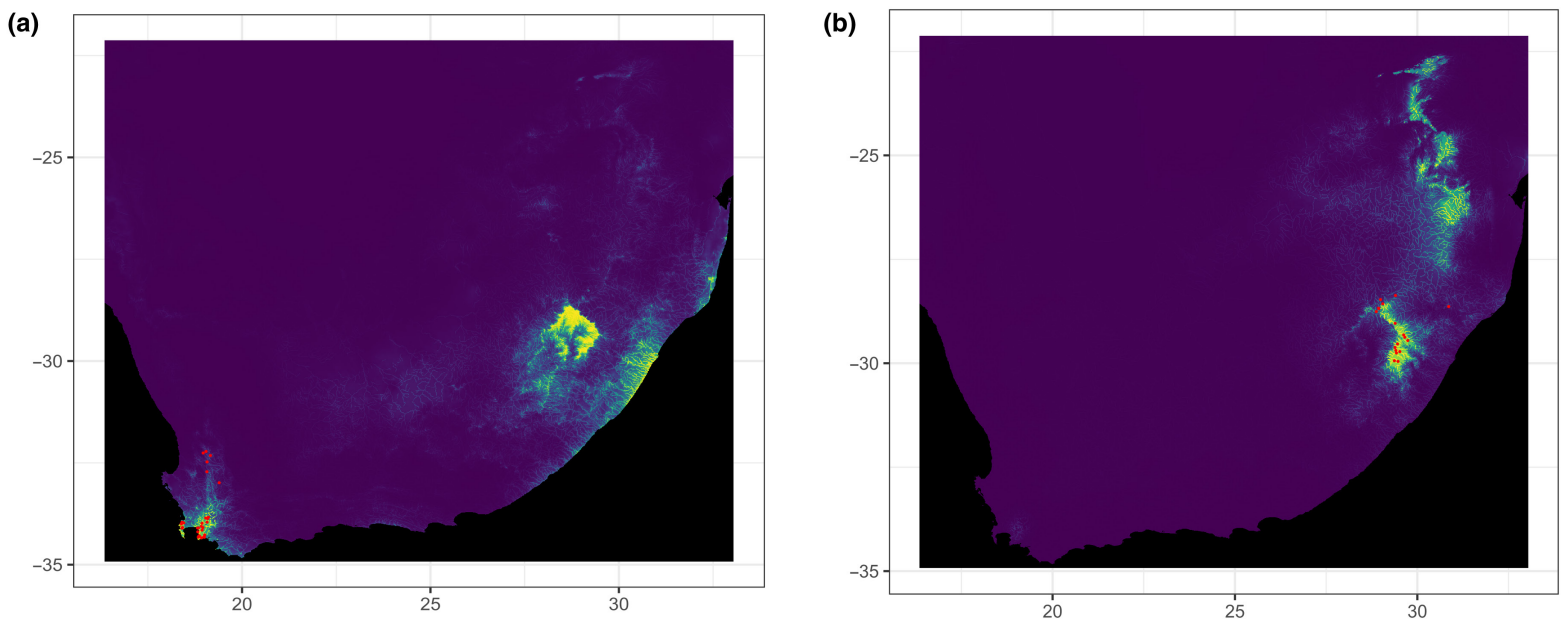
(a, b) Heatmaps indicating the continuous probability of climatic suitability for the western (Cape Fold Mountains – Clade A) and eastern (Drakensberg Mountains – Clade B) taxon groups.

The resulting variable contributions based on permutation importance (Table 7A,B) indicated that the distributions of the two taxon groups were differentially affected by climate. The CFM group (Clade A) was primarily influenced by temperature variables, specifically isothermality (Bio3) and temperature seasonality (Bio4), both of which indicate that an increase in temperature variability negatively impacts the distribution of this group. In contrast, the GE group (Clade B) was overwhelmingly influenced by the precipitation of the wettest quarter (Bio16), with precipitation being positively correlated with the likelihood of occurrence within this group. The SDMs, both visualised using continuous heatmaps (Figure 8a,b) as well as binary models generated using the maximum test sensitivity and specificity thresholds (results not shown) supported the results of the PCA, indicating minimal overlap between the climatic niches of the two taxon groups.

**TABLE 7:** The bioclimatic variable contribution for each of the two clades.

Variable	Percent contribution	Permutation importance
Western Mountains Clade (A)		
Bio13	59.4	0.6
Bio4	15.9	41.2
Rivers	13.8	6
Bio3	9.4	44.5
Bio15	1.1	5.4
Bio1	0.5	2.3
Eastern Mountains Clade (B)		
Bio16	81.8	89.5
Rivers	15.7	0.7
Bio6	1.9	7.4
Bio14	0.4	2.4
Bio4	0.2	0
Bio12	0.2	0

*Note*: Clade A representing the Cape Fold Mountains, while clade B represents the Drakensberg Mountains.

## 4 DISCUSSION

Our well‐resolved, time‐calibrated molecular phylogeny allows us to make inferences about the evolutionary patterns and processes in the montane living freshwater crabs and to decipher spatial and temporal drivers that catalysed cladogenesis (Figure 2). We observed a monophyletic montane living freshwater crab clade, comprised of two highly divergent main clades with the Cape Fold Mountain (CFM) and Great Escarpment (Drakensberg Mountain, GE) species forming clades A and B, respectively. Divergence time estimation within both major clades demonstrate that episodes of speciation are linked to Mio/Plio/Pleistocene pulses of tectonic uplift (based on the respective 95% HPD values obtained for the divergence time estimation, Figure 2) pronounced climatic ameliorations and landscape erosion, altering habitat and hydrological patterning resulting in fragmentation and the formation of novel habitat and niches into which lineages can radiate. Both clades were shown to occupy separate environmental niches and could be differentiated by climatic variables. In clade A, the main abiotic driver is temperature, specifically temperature seasonality, with the area being predominantly Mediterranean and characterised by warm dry summers and winter rainfall. In clade B, the main abiotic driver is rainfall, with the region being predominantly alpine temperate with summer rainfall and winter snowfall. Interestingly, while the inclusion of rivers systems as a categorical variable in model construction clearly impacted the predicted distribution of the focal taxa (Figure 8a,b), they received relatively low levels of permutation importance (Table 7A,B). However, this could be explained by high levels of correlation between the presence of rivers and the remaining continuous variables (such as rainfall and temperature seasonality).

Geographically, the Cape Fold Mountains are in close proximity to the cold Atlantic Ocean, while the Drakensberg Mountains are in close proximity to the warm Indian Ocean. In addition, the two mountainous blocks can also be differentiated based on altitude, with the Drakensberg Mountains being considerably higher compared to the Cape Fold Mountains (Cowling et al., 2009).

The ancestral species likely occurred at mid‐altitude regions during warmer, mesic periods during the Miocene prior to fragmentation, and were subsequently restricted to cooler, temperate, and higher altitude habitats under xeric conditions that dominated the Plio/Pleistocene (Cowling et al., 2009). Some important differences exist between the two clades. In clade A, sister species pairs are allopatric and confined to isolated CFM massifs. In contrast, in clade B, the GE (Drakensberg Mountains) *in situ* speciation and frequent sympatric lineages are present on the same mountain block (Figures 1 and 2). Furthermore, in the CFM (clade A), freshwater crab species were collected at considerably lower elevation (above sea level) in comparison to those from the GE (Drakensberg Escarpment, clade B), where species were exclusively associated with high altitudinal habitats (Table 1). Phylogenetically (Figure 2), both clades have sister species in the Eastern Cape mountains where the CFM and GE are in close geographic proximity, with *P. amathole*, from the Amathola Mountain range sister to western CFM species (clade A), while *P. baziya* from Baziya Forest is sister to GE species (Drakensberg Mountains) in clade B. Our unconstrained DECj model of range evolution analyses (Figure 3) indicated an ancestral dispersal in a north–south direction, originating in the Eastern Cape mountains, where both the CFM and GE acted as cradles and refugia. Phylogeographic studies of faunal assemblages in South Africa demonstrate an east‐south colonisation (Daniel et al., 2021; Maswanganye et al., 2017; Nielsen et al., 2018).

The exclusive application of the four species delimitation methods (ASAP, PTP, bGMYC, and STACEY) to the unilocus data (Figure 4a) retrieved between seven and 28 putative lineages, with limited congruence between methods (outgroups excluded). The latter results were generally incongruent with the existing taxonomic designations within the mountain clade of *Potamonautes*. Overall fine‐scale sampling, aided by the use of rapidly evolving mtDNA loci, frequently detects marked genetic structuring as a consequence of the isolation of conspecific populations to high altitude mountain habitat, restricting gene flow. Consequently, using these loci results in rapid population coalescence and marked genetic differentiation impacting the assignment of divergent populations to putative species clusters (Daniels et al., 2023). Intraspecific population genetic structure and a lack of shared maternal haplotypes have been documented among mountain crab species, corroborating limited dispersal (Phiri & Daniels, 2016; Wood & Daniels, 2016). Using the COI data from the present study, as evident from the large number of unconnected haplotypes and marked interspecific genetic ‘p’ distance values, corroborates a pattern of pronounced evolutionary divergence. Intraspecific ‘p’ distances range from <1% to as high as 3.68%. Additionally, the 138 COI sequences used during the present study retrieved 60 haplotypes, arranged in 12 distinct haploclusters with a minimum of one to nine missing or unsampled haplotypes and four haplotypes that were unconnected (S.R. Daniels, Unpublished data). Collectively, all the latter observations likely enhance the number of operational taxonomic units when subjecting the data to species delimitation.

However, the question remains as to which of the two hypotheses (MGH vs. MGSH) of mountain diversification is supported by our results. Differentiating between the two competing diversification hypotheses can be fraught with difficulty since it is difficult to demonstrate the adaptive value of characters. In both freshwater clades (A & B), there are recurring evolutionary themes with tectonic uplifts inducing climatic ameliorations, resulting in habitat fragmentation on the two separate mountain block types, in both the CFM and the GE. Our results favour the mountain gradient speciation hypothesis (MGSH) based on the fact that sister species with overlapping altitudinal distribution are in allopatry and not on the same mountain block. Our morphometric results (Figure 5) demonstrated a degree of overlap between five freshwater crab species suggesting some evidence for ecological adaptation or possible convergence is present among the species. Assuming the data is indicative of adaptation, the ultimate cause for these adaptations is very difficult to determine. The ecology, for example, diet, and habitat use and preference of freshwater crabs are poorly studied in South Africa, limiting ecological inferences. In fact, only two studies on the diet of large‐bodied riverine South African freshwater crabs (*P. perlatus* and *P. sidneyi*) are published (Hill & O'keeffe, 1992; Peer et al., 2015). Consequently, the feeding guilds among the mountain freshwater crab species are unknown; however, freshwater crabs are known to be dietary generalist and detrital feeders (Peer et al., 2015). Studies on the ecology and diet of high altitude mountain stream freshwater crabs are needed to understand their dietary intake. Morphologically, mountain living freshwater crabs are generally small‐bodied (carapace length < 30 mm, Gouws et al., 2000; Peer et al., 2023; Stewart, 1997), with smooth anterolateral carapace margins (with the exception *of P. parvispina*, which possesses a single small spine on the anterolateral carapace margins), and highly arched dactyli, suggesting some potential adaptation to the steep mountain stream gradient. However, disentangling the adaptive value of the morphological features is challenging and requires further scrutiny.

Within clade A, divergence was initiated during the late Miocene. The ancestral species in the CFM underwent episodic tectonic uplift of up to 150 and 300 m in the west and east, respectively (Partridge & Maude, 1987, 2000). Dramatic climatic oscillations, shifting from a predominant mesic environment during the Miocene to an increase in xeric conditions in the late Pliocene, were a consequence of the development of the proto‐Benguela upwelling current along the Western Cape coastline (Siesser, 1980; Siesser & Dingle, 1981). These climatic regimes resulted in the enhanced aridification of the interior of South Africa, that became more pronounced during the Pliocene, with intermitted small‐scale mesic shifts. During this period, a winter rainfall regime with prevalent summer droughts was established in the south‐western region of South Africa (Deacon et al., 1992). The resultant climatic shift produced a Mediterranean climatic regime that was markedly different compared to that of the more temperate summer rainfall experienced along the GEs, specifically in the Drakensberg Mountains. The Pleistocene observed intensified and continuous aridification cycles in the CFM region, significantly impacting freshwater habitat availability and resulting in its contraction to high altitude areas. Marine transgressions during the Pleistocene resulted in sea level increases of between 35 and 150 m higher than that of present, further resulting in mountain fauna being isolated and restricted to high altitude regions, impeding freshwater taxa dispersal (Partridge & Maude, 1987, 2000). Collectively, these tectonic uplifts, coupled with climatic oscillations promoted allopatric fragmentation of the ancestral species and its confinement to high altitude habitat. The Plio/Pleistocene arrival of several large‐bodied riverine freshwater crab species such as *P. barnardi*, *P. barbarai*, *P. perlatus*, and *P. danielsi* in lower riverine reaches in the Western and Eastern Cape likely resulted in interspecific competition limiting the dispersal capabilities of mountain dwelling freshwater crabs (Daniels et al., 2023). In the most recent phylogeny of *Potamonautes* based on additional DNA sequence data, *P. amathole* form the Eastern Cape, Amathola Mountains was retrieved sister to *P. parvispina* from the Cederberg and Kouebokkeveld Mountains, supporting an east–west ancestral connection (Daniels et al., 2023), to the exclusion of a cluster of sister species on the western CFM. It is noteworthy, that the phylogenetic reconstruction of velvet worms in *Peripatopsis* (an ancient lineage of euarthropoda) retrieved a sister taxon relationship between velvet worms from south‐eastern Cape forests to a species endemic to the Cederberg and Kouebokkeveld Mountains (Barnes et al., 2020). Similarly, Chakona et al. (2013) demonstrated a sister taxon relationship between the freshwater fish populations of *Galaxia* sp. from the southern Cape drainages and those from the Olifants river system that drains the confluence of the Cederberg Mountains. However, in the latter instance, the date of divergence was placed during the Pleistocene/Holocene, suggestive of continued connectivity between the south‐eastern Cape and the Cederberg and Kouebokkeveld Mountains. The Mio/Plio/Pleistocene triggered evolutionary radiations in several Cape lineages (Bentley et al., 2014; Daniel et al., 2020; Diedericks & Daniels, 2014; Engelbrecht et al., 2013; Galley et al., 2007; Linder & Hardy, 2005; McDonald & Daniels, 2012; Raphalo et al., 2020). Myburgh and Daniels (2022) suggest that Mio/Plio/Pleistocene climatic ameliorations together with barriers imposed by the CFM comprised the major drivers for cladogenesis in ectotherms. With respect to sampling intensity, CFM mountain areas in the Eastern Cape province require additional scrutiny to document their biodiversity.

In contrast, clade B (Drakensberg Mountains) underwent major episodes of in situ cladogenesis induced by geomorphic activity, since uplift was followed by erosion cycles which were particularly prevalent during the Mio/Pliocene epochs. Similar patterns of in situ radiations in the Drakensberg Mountain clades have also been reported in other faunal and floral taxa (Bentley et al., 2014; Galley et al., 2007; Travers et al., 2014). The Drakensberg Mountains underwent two periods of renewed tectonic uplift during the early Miocene and early Pliocene. These uplifts of between 150–300 and 600–900 m, respectively, raised the eastern margins of the GE (Knight & Grab, 2015). The escarpment plateau is thought to have extended towards the modern day coastline, and subsequent landscape erosion resulted in its present position in the interior of KwaZulu‐Natal (Knight & Grab, 2015). In clade B, we observe some evidence for erosion‐induced variation among lineages. Major bouts of cladogenesis in the Drakensberg Mountain clade (B) (3.40 Mya [2.05–5.00 Mya]; 3.1 Mya [1.63–4.06 Mya]) are consistent with post uplift scarp erosion. Similar bouts of speciation have been reported among the paper daisies (*Macowania* sp.) (Bentley et al., 2014). Pronounced allopatric divergence induced via tectonic uplift, erosion, climatic amelioration, and drainage rearrangements followed by subsequent expansion events resulted in species occurring in sympatry in some clades. Knight and Grab (2018) suggest that uplift together with climate factors such as precipitation and weathering are drivers of drainage evolution along the Drakensberg Mountains. However, drainage evolution is complex and its impact on the partitioning of aquatic diversity is poorly understood. The Plio/Pleistocene divergence of the large‐bodied riverine congeners, *P. danielsi* and *P. sidneyi*, and their occurrence at lower altitudes along the GE, likely enhanced ancient interspecific competition, further restricting the distribution of the small‐bodied mountain crab ancestral species.

In the main Drakensberg Mountain escarpment, we observe three major clades, sister to *P. baziya* from the Eastern Cape province. These three major clades include a basal clade comprised of *Potamonautes* sp. nov. 1, present in the central Injasuthi, Impendle, Karkloof, Dargle forest, and Cathedral Peak localities (Figure 1). The second clade comprising *P. clarus* is present in the northern Drakensberg Mountain escarpment at Gudu Falls, Oliviershoek Pass, Mahai, and Himeville NR and extends into the interior as far as Quadeni Forest, while the third clade is widely distributed in the central Drakensberg representative of *P. depressus* and includes the localities Himeville, Kamberg, Vergelegen NR, Sani Pass, Cathedral Peak, Garden Castle NR, Coleford NR, Cobham, and Monks Cowl (Figure 1). The presence of two highly divergent clades in the central Drakensberg Mountain range provides evidence for the landscape erosion scenario advocated by other research groups (Bentley et al., 2014; Galley et al., 2007; Travers et al., 2014). At four sample localities, Injasuthi, Cathedral Peak, Monks Cowl, and Himeville, we observe highly divergent lineages that are either the result of at least two independent colonisation events or secondary contact between previously isolated lineages. The application of the four species delimitation methods delineated an inconsistent number of molecular OTUs in the Drakensberg Mountain clade. Phiri and Daniels (2016) estimated the presence of six novel lineages in the *P. clarus*/*P. depressus* complex. The exclusive reliance on mtDNA to delineate evolutionary lineages in the clade B (main Drakensberg Mountain lineage) (Figure 4c) revealed between five and 20 putative species. By contrast, the addition of the three nuDNA loci (Figure 4b) revealed between two and six lineages, while the combined mt + nuDNA data revealed between one and 48 lineages (Figure 4d) (all excluding the two outgroup species). The new clade, *Potamonautes* sp. nov. 1 (represented by Injasuthi specimens in the species delimitation analyses) is detected to a varying degree, frequently shown to comprise at least two novel lineages (Figure 4b–d), whereas the application of the species delimitation methods to the mtDNA data with larger sample sizes (Figure 4a), retrieved one to six putative units. When specifically exploring the application of nuDNA markers including 28S rRNA, DecapANT, and PEPCK, we observed fewer OTUs due to the slow evolutionary rate of nuDNA loci, limiting differentiation exclusively derived from these genetic markers. In addition, the differences between the mt and nuDNA for the Drakensberg Mountains clade can also partially be attributed to the differences in gene and species trees obtained from the two datasets. The uncorrected ‘p’ COI distance values for *Potamonautes* sp. nov. 1 are similar or higher when compared to distance values reported in other freshwater crab sister species, further supporting the genetic divergence of this lineage (Daniels et al., 2014, 2023; Daniels & Bayliss, 2012; Daniels & Klaus, 2018; Phiri & Daniels, 2014). Our approach is a more conservative one, since there is a blatant oversplitting of lineages in clade B that can be attributed to the deep divergence and marked genetic variation among conspecific populations due to a high degree of isolation. We advocate for an integrative approach, including the use of traditional morphological characters of the carapace, gonopods (one and two), and maxillipeds, when delineating lineages.

The new freshwater crab species from the central Drakensberg Mountains (*Potamonautes* sp. nov. 1) will be described in a future publication (S. R. Daniels, personal communications). The eastern Drakensberg Mountain escarpment as it extends northwards into the Limpopo and Mpumalanga provinces and westwards into the Maluti Highlands, in neighbouring Lesotho, and the southwards into Eastern Cape province requires additional sampling efforts to document the biodiversity patterning of freshwater crabs since high altitude areas in the latter regions are poorly sampled and potentially harbouring additional undocumented freshwater crab species. The present study represents an important advance in our understanding of the mechanisms responsible for divergence in temperate mountain dwelling invertebrate fauna in South Africa. Furthermore, it highlights the role of tectonic uplift and climatic oscillations as major drivers of cladogenesis among freshwater lineages.

## AUTHOR CONTRIBUTIONS

**Savel R. Daniels:** Conceptualization (lead); data curation (lead); investigation (lead); project administration (lead); supervision (lead); writing – original draft (lead); writing – review and editing (lead). **Nasreen Peer:** Writing – original draft (supporting); writing – review and editing (supporting). **Angus Macgregor Myburgh:** Methodology (supporting); software (supporting); writing – original draft (supporting); writing – review and editing (supporting). **Aaron Barnes:** Data curation (supporting); formal analysis (supporting); methodology (supporting); software (supporting); writing – original draft (supporting); writing – review and editing (supporting). **Sebastian Klaus:** Data curation (supporting); formal analysis (supporting); methodology (supporting); writing – original draft (supporting); writing – review and editing (supporting).

## CONFLICT OF INTEREST STATEMENT

No conflict of interest to declare.

## Data Availability

I declare the above at the end of the paper, Genetic data (nxs) and the niche modelling data.
